# A Probable Catastrophic Antiphospholipid Antibody Syndrome/Thrombotic Storm Presenting As Rapidly Evolving Multifocal Ischemic and Hemorrhagic Strokes: A Case Report

**DOI:** 10.7759/cureus.35584

**Published:** 2023-02-28

**Authors:** Mohammad Abu-Abaa, Ghassan Al-Qaysi, Sindhu Chadalawada, Adedeji Cole

**Affiliations:** 1 Internal Medicine, Capital Health Regional Medical Center, Trenton, USA

**Keywords:** catastrophic antiphospholipid antibody syndrome (caps), deep vein thrombosis (dvt), stroke, lupus anticoagulant, antiphospholipid antibody syndrome (aps), thrombotic storm, catastrophic antiphospholipid antibody syndrome

## Abstract

Catastrophic antiphospholipid antibody syndrome (CAPS) is a life-threatening disorder. It is a rare and severe form of antiphospholipid antibody (APL) syndrome characterized by widespread multisystemic thrombosis. We present a 55-year-old male patient with acute cerebellar hemorrhagic stroke who developed widespread progressive microthrombosis and macrothrombosis manifesting as progressive bilateral ischemic strokes with lower extremities deep vein thrombosis (DVT) and acute renal failure within a week of presentation. The diagnosis and initiation of therapy were established after serological confirmation. This case adds to a limited number of cases of CAPS in literature and is interesting given the rarity of CAPS and thrombotic storm (TS) as well as the lack of inciting factor triggering CAPS/thrombotic syndrome. This case also helps to remind the clinicians of the importance to consider CAPS, even prior to serological confirmation, in those with rapidly progressive thrombotic events, as delayed diagnosis and therapy can yield poor clinical outcomes.

## Introduction

Antiphospholipid antibody syndrome (APL) is a multisystemic autoimmune syndrome characterized by arterial and venous thrombosis and/or pregnancy loss with persistently positive antiphospholipid antibodies, including lupus anticoagulant (LA), anti-beta 2 glycoprotein I and anticardiolipin antibodies [1]. Both thrombotic and non-thrombotic manifestations can be seen [1]. Catastrophic APL (CPAS) is the most severe type characterized by multiorgan involvement, usually by microthrombosis, over a short period of time [1]. The most commonly affected organs are the central nervous system (CNS), kidneys, lungs, and gastrointestinal tract (GIT) [2].

## Case presentation

**Figure 1:**
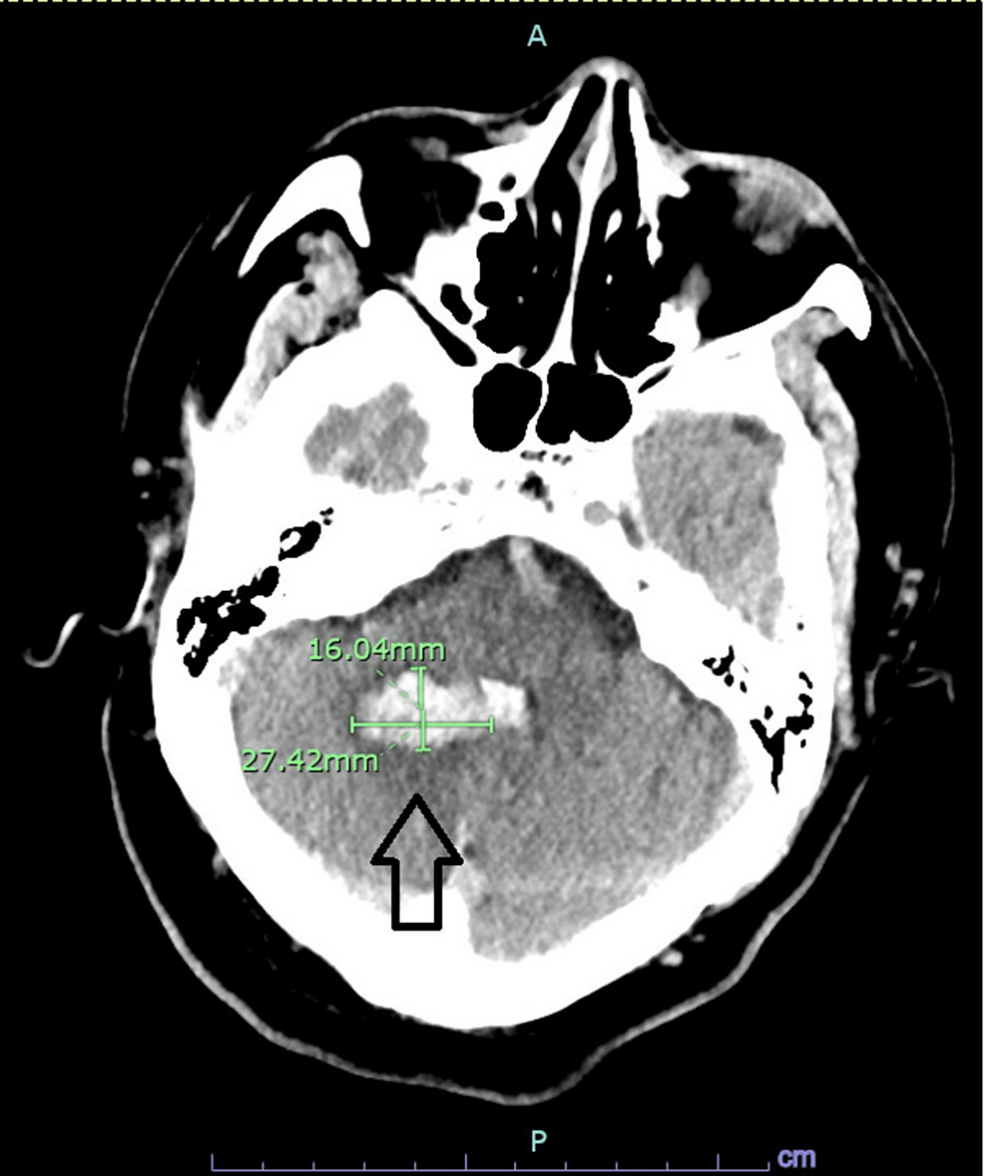
CT head Computed tomography (CT) head without contrast showing evidence of acute right-sided cerebellar hemorrhage affecting the superior cerebellar peduncle with extension to the fourth ventricle, left-sided displacement of the cerebral aqueduct and surrounding cerebral edema without hydrocephalus (arrow)

**Figure 2:**
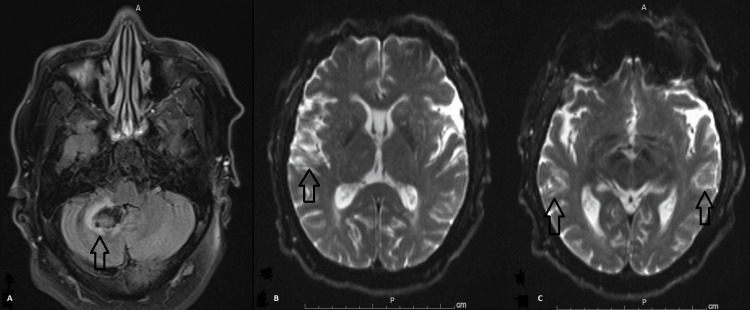
Initial MRI brain Magnetic resonance imaging (MRI) brain with and without contrast showing right-sided cerebella hemorrhagic stroke (arrow in A), bilateral cerebral hemisphere acute/sub-acute infarctions mainly affecting watershed areas, centrum semiovale, and corona radiata (arrows in B and C)

**Figure 3:**
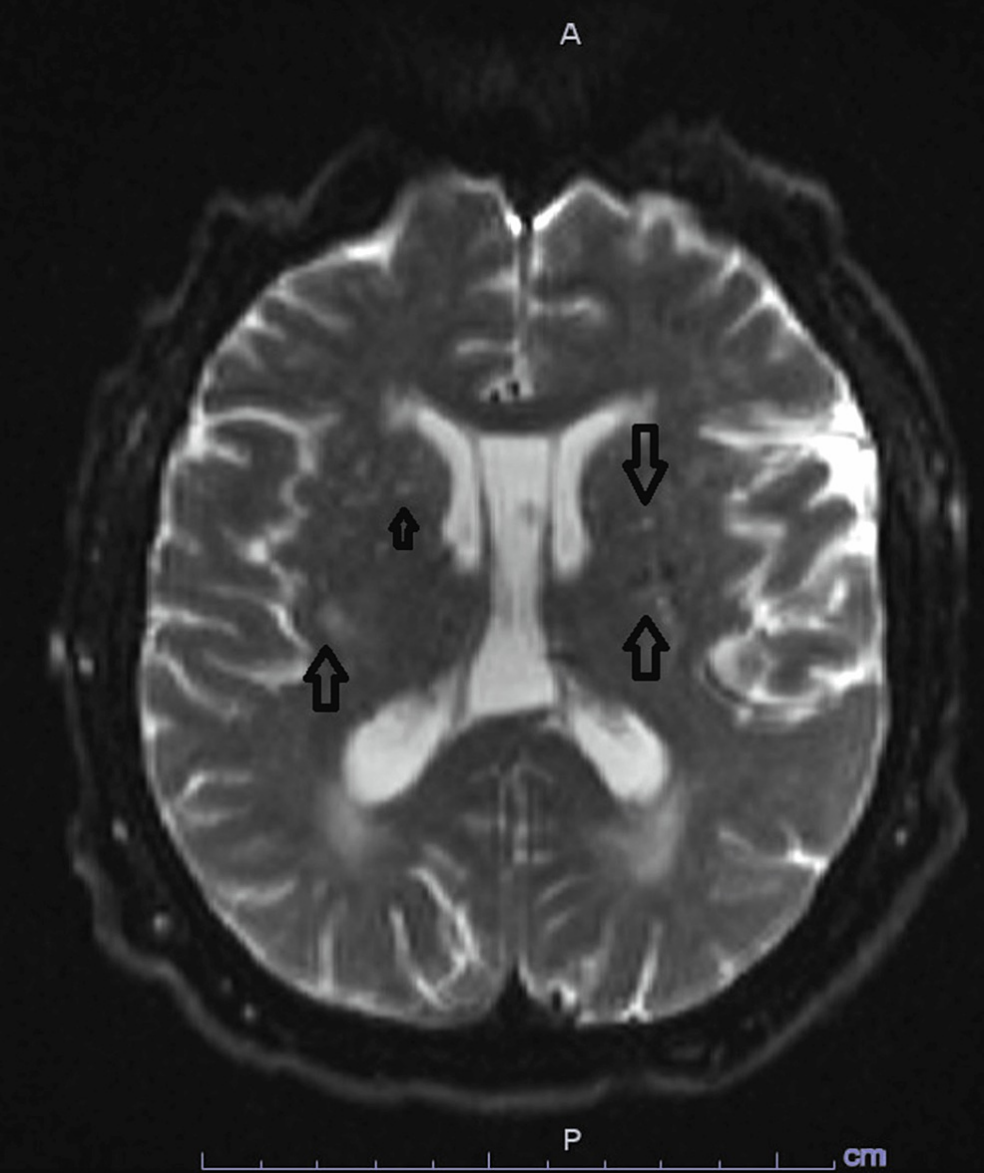
Initial MRI brain with cerebral strokes Magnetic resonance imaging (MRI) brain with and without contrast further delineating the extent of bilateral cerebral hemisphere acute/sub-acute infarctions mainly affecting watershed areas, centrum semiovale, and corona radiata (arrows)

A 55-year-old male patient presented to the emergency department (ED) with sudden onset repeated vomiting and tingling of the lower extremities a few hours prior to presentation. Past medical history was remarkable only for hypertension, hyperlipidemia, and alcohol use disorder. There was no reported preceding fever, diarrhea, cough, abdominal pain, and no history of illicit drug use. In the ED, the patient was pale-looking, diaphoretic and hypertensive. Vitals signs included a blood pressure of 180/100 mmHg, heart rate of 98 beats per minute, respiratory rate of 22 cycles per minute, SpO2 of 94% on room air, and temperature of 36.6 degrees Celsius. On physical examination, he was lethargic but arousable and fully oriented, scanning speech with mild dysarthria, left-sided gaze deviation with the inability to overcome midline and right-sided nystagmus on attempted right gaze, subtle left nasolabial fold flattening, symmetrical, nondilated and reactive pupils at 3 mm, muscle power of 4/5 all over and intact light touch grossly with right upper extremity ataxia. Computed tomography (CT) head without contrast showed evidence of acute right-sided cerebellar hemorrhage affecting the superior cerebellar peduncle with extension to the fourth ventricle, left-sided displacement of the cerebral aqueduct and surrounding cerebral edema without hydrocephalus (Figure 1). No evidence of vascular stenosis or occlusion, aneurysm, or arteriovenous malformation was evident on CT angiography of the head and neck. Magnetic resonance imaging (MRI) brain with and without contrast showed bilateral cerebral hemisphere acute/sub-acute infarctions mainly affecting watershed areas, centrum semiovale, and corona radiata with evidence of chronic microhemorrhages (Figures 2, 3). Basic lab workup showed only leukocytosis at 16,000 cells/ml with normal coagulation profile including prothrombin time (PT), partial thromboplastin time (PTT), and international normalized ratio (INR) and fibrinogen level. Renal function was also intact. COVID-19 polymerase chain reaction (PCR) was negative. Nicardipine, as well as hypertonic saline infusion, were started.

The absence of vascular abnormalities contributing to the insult, including signs of vasculitis and early venous drainage, was confirmed by a diagnostic cerebral angiogram. Continuous electroencephalography (EEG) showed generalized slowing with no seizure. EKG and telemetry monitoring showed sinus rhythm. Transthoracic and transesophageal echocardiogram showed no evidence of a cardioembolic source, including no intracardiac thrombus and vegetation, negative bubble study, and no aortic plaque. Daily CT head showed stability of intracranial hemorrhage (ICH). However, his mental status was declining, and he was increasingly more obtunded with a Glasgow coma scale of three, requiring intubation. Erythrocyte sedimentation rate (ESR) was elevated at 104 U/L (reference range 0-20 U/L) with an elevation of C-reactive protein (CRP) at 8.9 mg/dl (reference range less than 1 mg/dl). Platelet count remained stable throughout hospitalization. No schistocytes were seen. PTT transiently increased as high as 54 seconds (reference 24-36 seconds). PT and INR remained within normal limits. Cerebrospinal fluid (CSF) analysis showed xanthochromia with elevated WBC 291 cells/ml with lymphocyte predominance, elevated protein at 112 mg/dl, and normal glucose at 56 mg/dl. The Meningitis/encephalitis panel was negative. CSF, blood, and urine cultures remained negative.

**Figure 4:**
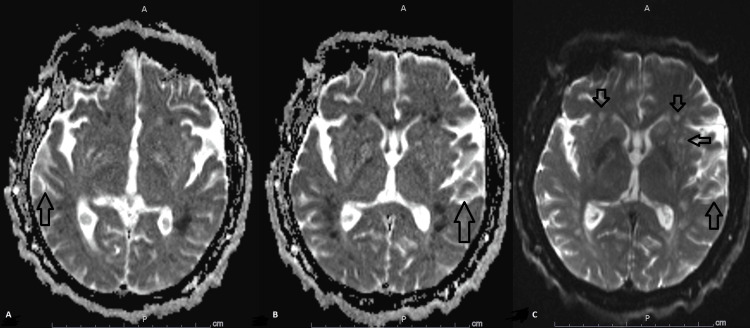
Diffusion trace MRI brain Diffusion trace MRI brain sequence showing the progression of cerebral infarctions (arrows in A, B, and C) and bilateral centrum semiovale, corona radiata, periventricular and gangliocapsular areas as well as corpus callosum (arrows in C)

**Figure 5:**
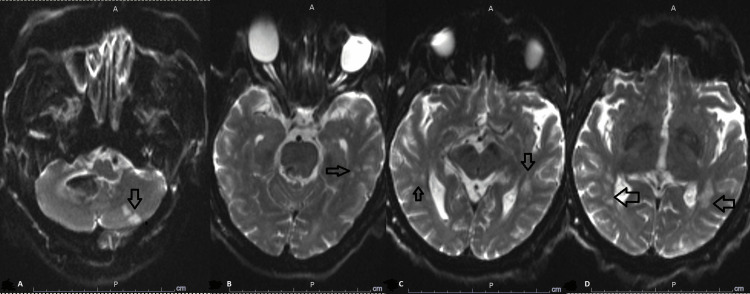
Subsequent diffusion trace MRI brain Diffusion trace MRI brain showing a new left-sided cerebellar infarction (arrow in A) with new punctate bilateral infractions affection cerebral hemispheres (arrows in B, C, and D)

**Figure 6:**
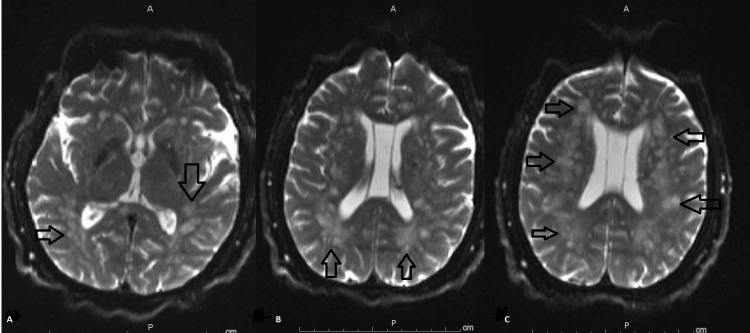
Further delineation of ischemic strokes on diffusion trace MRI brain Further delineation of the extent of punctate ischemic strokes on diffusion trace MRI brain involving cerebral hemispheres (arrows in A, B, and C)

**Figure 7:**
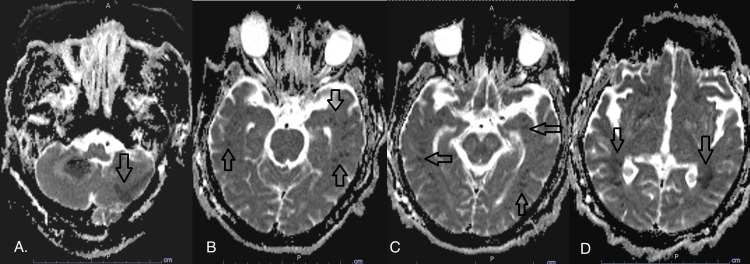
MRI brain diffusion-ADC window MRI brain diffusion-apparent diffusion coefficient (ADC) window showing new left-sided cerebella stroke (arrow in A), with the progression of cerebral strokes (arrows in B, C, and D)

**Figure 8:**
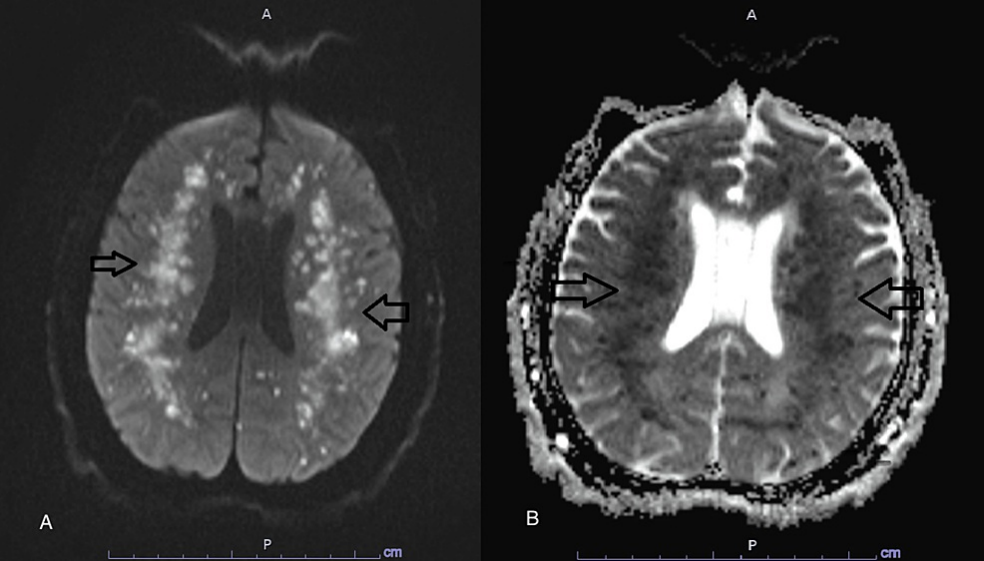
Diffusion trace and diffusion-ADC MRI A is a diffusion trace MRI showing the extensive white matter strokes (arrows), and B is a diffusion-apparent diffusion coefficient (ADC) window showing corresponding defects (arrows)

On day five of hospitalization, he developed acute kidney injury with an abrupt increase in serum creatinine from 0.78 mg/dl to 2.51 mg/dl. This did not significantly improve with volume expansion. Although a repeat MRI brain showed stability of intracerebral brain hemorrhage (ICH) with its intraventricular hemorrhage (IVH), it showed progression of cerebral infarctions to affect bilateral centrum semiovale, corona radiata, periventricular and gangliocapsular areas as well as corpus callosum (Figure 4). Evidence of bilateral lower extremities acute proximal and distal deep venous thrombosis (DVT) was also appreciated. An inferior vena cava filter was placed. Three days later, a repeat MRI brain showed a new left-sided cerebellar infarction with new punctate bilateral infractions affection the cerebral and cerebellar hemispheres (Figures 5, 6, 7, 8). Anticardiolipin IgM antibody was elevated at 97 unit/ml (reference less than 12 unit/ml) with normal anticardiolipin IgA and IgG, positive lupus anticoagulant (PTT-lupus anticoagulant 75.8 with reference less than 51.9 seconds), normal anti-beta 2 glycoprotein IgA, IgG and IgM antibodies, negative ANA, negative homocysteine, negative Factor V Leiden and prothrombin mutations, normal qualitative and quantitative protein C and S levels and normal antithrombin 3. P and C anti-neutrophil cytoplasmic antibodies (ANCA) were negative; the ADAMTS13 level was less than 5%.

CT abdomen, pelvis, and chest did not show additional areas of infarction or suspicious findings for malignancy. He was then started on five days of high-dose pulse corticosteroid therapy followed by a slow taper. No clinical improvement was attained, and tracheostomy with percutaneous endoscopic gastrostomy was pursued. Lupus anticoagulant remained positive after three months with no further clinical improvement.

## Discussion

Catastrophic antiphospholipid antibody syndrome (CAPS) is an accelerated form of antiphsopholipid antibody (APL) syndrome. Approximately 75% of cases of CAPS are seen in women [3]. I can affect individuals from 11 to 60 years of age. Only 29 cases of CAPS were reported in those older than 60 years [3]. It accounts only for 1% of cases of APL syndrome [3]. Therefore, clinicians tend to not include this initially in the differential diagnosis of a new onset thrombotic event, especially in the clinical context of no prior triggering factor or rheumatological history. The prevalence of APL antibodies in the general population is 1-5% [4]. Therefore, positive APL does not establish the diagnosis. 

About 50% of patients with CAPS have no history of APL syndrome [4]. The clinical spectrum of APL includes APL antibodies positivity with no clinical events, APL antibodies positivity with non-criteria manifestations (including hemolytic anemia, thrombocytopenia, cardiac valves involvement, and nephropathy), APL syndrome with criteria-based manifestation (including arterial and venous thrombosis and pregnancy loss), and CAPS. APL and CAPS can also present with predominant hematological manifestations, including thrombocytopenia, hemolytic anemia, and disseminated intravascular coagulopathy (DIC), which leads to overlap with other thrombotic microangiopathies (TMA), and this was named microangiopathic APL [5]. Severe thrombocytopenia is uncommon [2]. DIC can complicate one-third of those with CAPS [6]. Renal involvement is characterized by a more than 50% increase in creatinine, proteinuria more than 0.5 g/day, and severe hypertension of more than 180/100 mmHg or combination [6]. Central nervous system (CNS) involvement is seen in 62% of cases and most commonly includes hypertensive encephalopathy, ischemic encephalopathy, stroke, and cerebral venous thrombosis [6]. Neurological involvement is the second most common at presentation after pulmonary involvement [7]. 

Triggering factors of CAPS include infections, anticoagulant withdrawal, medications (including thiazides, oral contraceptives, captopril, danazol), obstetric complications, malignancy, and SLE flare [3,7]. The pathogenic mechanism of APL/CAPS involves four interrelated mechanisms, including cell activation, fibrinolysis inhibition, anticoagulation inhibition, and complement activation [2]. 

Although transient positivity of APL antibodies can be seen in infections and persistent positivity of APL antibodies over a 12-week period is crucial for the diagnosis, positive LA is more predictive of APL-associated thrombosis, especially if tested off of anticoagulation [1]. Definitive CAPS diagnosis requires four of the following, dysfunction of three or more organs, rapid progression within less than a week, microthrombosis on histological examination, and positive APL antibodies. This is based on the 2003 international task force guidelines for the diagnosis of definite and probable CAPS [1,8]. Our case met three out of these criteria (with arterial thrombosis resulting in multifocal ischemic strokes, venous thrombosis resulting in bilateral extensive lower extremities thrombosis, acute kidney failure with no evidence of pre-renal or postrenal components that is likely explained by microangiopathy, rapid progression within a week of onset and positive LA antibody). This indicates probable CAPS. DIC was unlikely as the coagulation profile and fibrinogen were normal on presentation. Other thrombotic etiologies were ruled out including infection, heparin-induced thrombocytopenia (HIT), and thrombotic thrombocytopenic purpura (TTP). No thrombosis risk factors were noted in the case. A limitation of our case is the lack of histological examination that was not pursued, given the severity of the neurological insult. 

Thrombotic storm (TS) is a clinical syndrome that is characterized by multifocal severe thrombotic events. It is based on clinical diagnosis and can be caused by several prothrombotic disorders, including CAPS [9]. Macrovascular thrombosis is more predominant than microvascular thrombosis in thrombotic storm as compared to CAPS, as in this case [10]. Although our patient is more than 50 years old, other clinical characteristics of TS were seen, including more than two acute arterial and venous thromboembolic events within a one-week duration with progressive nature [10]. 

Treatment should not be delayed awaiting lab confirmation if clinical suspicion is high. Treatment is directed to suppress the cytokine cascade and also against thrombotic events [4]. Therapeutic options include corticosteroids, anticoagulation, plasma exchange, intravenous immunoglobulin (IVIG), and cyclophosphamide [3]. In our patient, the presence of ICH precluded consideration of anticoagulation, and the extent of neurological damage also precluded consideration of aggressive immunosuppression. The prognosis remains poor, although the mortality rate has been reduced from 53% to 37% with the current therapeutic approaches [11].

## Conclusions

CAPS is a rare entity and can be the first presentation of APL syndrome. The lack of a triggering factor does not preclude the diagnosis. The occurrence of multifocal arterial and venous thromboses over a short period of time, as in this case, should raise the suspicion of CAPS. In the presence of high clinical suspicion, therapy should be commenced without waiting for lab confirmation. This is especially important as early recognition and treatment have been reported to improve the outcome. A multidisciplinary approach to the diagnosis and treatment is recommended. The clinical distinction of CAPS from other TMA, including DIC, TTP, and HIT, is challenging as clinical and lab findings tend to overlap.
